# Hyoscine N-butylbromide administration before colostrum feeding improves passive transfer in dairy calves

**DOI:** 10.3389/fvets.2026.2002840

**Published:** 2026-10-14

**Authors:** Durmuş Fatih Başer, Abdurrahman Kürşat Özsoy

**Affiliations:** Department of Internal Medicine, Faculty of Veterinary Medicine, Afyon Kocatepe University, Afyonkarahisar, Türkiye

**Keywords:** colostrum, hyoscine N-butylbromide, immunoglobulin G, intestinal motility, neonatal calf, passive transfer

## Abstract

This study evaluated whether hyoscine N-butylbromide administration before colostrum feeding could influence colostral immunoglobulin G (IgG) absorption and passive transfer in neonatal Jersey calves. Eighteen clinically healthy calves were allocated to a control group (*n* = 6) or a hyoscine N-butylbromide-treated group (*n* = 12). Treated calves received hyoscine N-butylbromide at 0.4 mg/kg intramuscularly 15–30 min before the first colostrum feeding. All calves received high-quality maternal colostrum (Brix ≥25%) within 6 h after birth. Serum Brix was measured at 48 h, and the same serum samples were analyzed for IgG, total protein, gamma-glutamyl transferase (GGT), and glucose. Treated calves had higher serum Brix than controls at 48 h (10.96 ± 0.81 vs. 9.53 ± 0.7%; *p* = 0.002). Serum IgG was also higher in treated calves at 48 h (3,243 ± 794 vs. 2,175 ± 1,150 mg/dL; *p* = 0.034), whereas total protein, GGT, and glucose did not differ between groups (*p* ≥ 0.217). These preliminary findings suggest that hyoscine N-butylbromide administration before colostrum feeding may be associated with improved passive transfer indicators in neonatal calves; however, confirmation in larger, adequately powered studies is required.

## 1 Introduction

The transfer of maternal immunoglobulins across the placenta in cattle is prevented by the synepitheliochorial placental structure, which consists of six tissue layers separating the maternal and fetal circulations (1). As a result of this physiological barrier, calves are born agammaglobulinemic and become entirely dependent on colostrum intake after birth to acquire systemic immunity (2, 3). In addition, bovine colostrum contains immunoglobulins and several bioactive constituents that contribute not only to passive immunity acquisition but also to early postnatal immune and metabolic adaptation in neonatal calves (4, 5). The success of transfer of passive immunity (TPI) depends not only on the quality, quantity, and timing of colostrum feeding but also on the absorptive capacity of the neonatal intestine (6, 7). Failure of passive transfer has been associated with a higher risk of morbidity and mortality in neonatal calves and may also contribute to economic losses in dairy production systems. Differences in colostrum handling and management practices have been shown to contribute to variability in passive immunity transfer success among dairy farms (8). On-farm Brix refractometry has also been widely used as a practical method for estimating colostrum IgG concentration and supporting colostrum management decisions (9).

Immunoglobulin G (IgG) absorption in neonatal calves is a complex process mediated by enterocytes of the small intestine and involves non-specific pinocytosis, intracellular processing, and transcytosis into circulation (10, 11). In addition, the neonatal Fc receptor (FcRn) has been reported to contribute to the protection and transport of IgG across epithelial cells and to the regulation of its persistence in circulation (12, 13). However, this absorptive capacity rapidly declines within the first 24–36 h after birth due to intestinal closure, which markedly limits the absorption of macromolecules (2).

Despite standardized colostrum management practices, substantial variation in apparent absorption efficiency (AEA) has been reported among calves (14). This indicates that IgG absorption cannot be explained solely by colostrum characteristics and that additional factors related to neonatal intestinal physiology may influence the process. Gastrointestinal motility is one of the physiological mechanisms that determines the rate at which luminal contents move through the intestine and may affect the contact time between colostral immunoglobulins and the intestinal epithelium. It has been suggested that increased luminal residence time may enhance absorption efficiency by increasing the interaction of IgG with enterocytes and FcRn-mediated transport mechanisms (15).

Within this physiological framework, pharmacological modulation of intestinal motility may represent a potential adjunctive approach for influencing passive immunity acquisition. Hyoscine N-butylbromide (Buscopan) is an anticholinergic spasmolytic agent that reduces gastrointestinal smooth muscle activity by blocking muscarinic receptors (16–18). Veterinary evidence in calves also supports a gastrointestinal effect of butylscopolamine-containing treatment; in calves with experimentally induced nutritional diarrhea, administration of a hyoscine N-butylbromide–dipyrone combination reduced fecal and fecal water output (19). However, pharmacological reduction of gastrointestinal motility in neonatal calves should be approached cautiously, as impaired luminal clearance may theoretically favor retention of bacteria and their products within the gastrointestinal tract (20).

Through this mechanism, it may decrease peristaltic activity and alter luminal transit dynamics. However, studies investigating the relationship between intestinal motility and nutrient or molecule absorption have reported variable results, suggesting that the effect depends on the characteristics of the absorbed substance and the physiological conditions (21, 22). In particular, the effect of pharmacological modulation of intestinal motility on the absorption of high-molecular-weight macromolecules such as colostral IgG has not been clearly evaluated. Previous findings under standardized colostrum management conditions have shown that serum IgG concentrations may vary despite comparable colostrum quality, suggesting differences in apparent absorption efficiency among calves (23).

In the existing literature, strategies aimed at improving passive transfer in neonatal calves have primarily focused on colostrum management, whereas intestinal motility has received limited attention as a potential physiological factor affecting IgG absorption. Therefore, the objective of this study was to evaluate the effect of hyoscine N-butylbromide administration before the first colostrum feeding on passive transfer indicators in neonatal Jersey calves. Serum IgG concentrations and serum Brix values were used to assess passive immunity acquisition. It was hypothesized that hyoscine N-butylbromide administration before colostrum intake would be associated with higher serum IgG concentrations and serum Brix values by potentially influencing gastrointestinal transit dynamics.

## 2 Materials and methods

### 2.1 Animals and experimental design

This study was conducted under standard management and environmental conditions on a single commercial dairy farm (Tarım Kredi Hayvancılık A.Ş., Kırklareli, Türkiye). The study was conducted during April and May. A total of 18 clinically healthy neonatal Jersey calves of both sexes, born from cows in their third lactation, were enrolled immediately after birth and before first colostrum feeding. No cases of dystocia were observed during the study period. All sampling procedures were carried out within a short time frame under similar environmental conditions to reduce the potential effect of seasonal variation. The first 12 eligible calves were alternately allocated to the control and hyoscine N-butylbromide-treated groups, resulting in 6 calves in each group. Subsequently, six additional eligible calves enrolled within the study period were assigned to the treatment group, resulting in final group sizes of 6 control calves and 12 treated calves. The animal study protocol was approved by the Local Animal Experiments Ethics Committee of Afyon Kocatepe University (AKUHADYEK), Afyonkarahisar, Türkiye (protocol code AKUHADYEK-115-24; approval date: 29 January 2024).

### 2.2 Hyoscine N-butylbromide administration

Calves in the treatment group received a single intramuscular dose of 0.4 mg/kg hyoscine N-butylbromide (Buscopan®, Boehringer Ingelheim, Ingelheim am Rhein, Germany) 15–30 min before the first colostrum feeding. Calves in the control group received no pharmacological treatment before colostrum feeding.

### 2.3 Colostrum management and feeding protocol

Immediately after birth, calves were separated from their dams before suckling and placed in individual pens. Colostrum was obtained from each calf’s own dam at first milking, and only calves born to cows producing high-quality colostrum, defined as Brix ≥25%, were included in the study. Colostrum Brix values were comparable between groups at enrollment.

Each calf received colostrum at a total volume corresponding to 10–12% of body weight within the first 6 h after birth using a bottle. The first colostrum feeding, corresponding to approximately 5–6% of body weight, was administered within the first 30 min after birth, and the remaining 50% of the total colostrum volume was administered within the subsequent 6 h. The source, volume, and timing of colostrum feeding were standardized across calves to minimize colostrum-related variation. After colostrum administration, calves were fed milk according to the farm’s standard feeding protocol.

### 2.4 Dry period feeding and vaccination protocol

All cows included in the study were managed under the same dry-period feeding program. The diet consisted of straw, alfalfa hay, wheat silage, corn silage, and a commercial dry cow concentrate. The ration contained approximately 49.4% dry matter, 11.8% crude protein on a dry matter basis, and 51.6% amylase-treated neutral detergent fiber corrected for organic matter (aNDFom), with a forage proportion of approximately 72.2%. Net energy for lactation was formulated at 1.27 Mcal/kg dry matter. The dietary cation-anion difference, calculated as [Na + K] − [Cl + S], was maintained at approximately +10.4 mEq/100 g dry matter. A commercial mycotoxin binder (Biomin®, Biomin GmbH, Getzersdorf, Austria) was included in the ration. All cows were vaccinated twice between days 210 and 255 of gestation with a combined vaccine containing rotavirus, coronavirus, and *Escherichia coli* K99 antigens (Kolibin® RC, Bioveta a.s., Ivanovice na Hané, Czech Republic), according to the manufacturer’s recommendations.

### 2.5 Sample collection and preparation

Colostrum samples were collected at the first milking before being fed to the calves. Colostrum quality was determined using a digital Brix refractometer (PAL-1, ATAGO Co., Ltd., Tokyo, Japan). A total of 20 mL of colostrum was collected from each sample and stored at −20 °C until analysis. Blood samples were collected from the jugular vein of calves at 48 h after birth. Samples were centrifuged at 3000 rpm for 5 min to obtain serum, and serum samples were stored at −20 °C until analysis.

### 2.6 Laboratory analyses

Serum immunoglobulin G (IgG) concentrations were determined using the radial immunodiffusion (RID) method (Triple J Farms, Washington, USA; Lot: 7284A65), according to the manufacturer’s instructions. Serum Brix values were measured using a digital Brix refractometer (PAL-1, ATAGO Co., Ltd., Tokyo, Japan). Serum total protein concentrations, gamma-glutamyl transferase (GGT) activity and glucose concentrations were measured using an automatic biochemical analyzer (Mindray BS-300, Mindray Bio-Medical Electronics Co., Shenzhen, China) with standard enzymatic methods.

### 2.7 Statistical analysis

Statistical analyses were performed using Minitab 18 software (Minitab Inc., State College, PA, USA). Normality and homogeneity of variances were assessed using the Ryan–Joiner and Levene’s tests, respectively. Continuous variables were compared between groups using independent-samples t-tests assuming equal variances, except for serum total protein, which was analyzed using Welch’s independent-samples t-test because of heterogeneity of variance. Sex distribution was compared using Fisher’s exact test. The timing of the first colostrum feeding was summarized descriptively because all calves received their first feeding within 30 min after birth. Maternal colostrum Brix values are presented as mean ± SD, serum outcome variables as mean ± SD, and categorical variables as n (%). All tests were two-sided, and statistical significance was declared at *p* < 0.05.

## 3 Results

As shown in Table 1, maternal colostrum Brix values were comparable between the control and hyoscine N-butylbromide-treated groups (29.22 ± 3.58% vs. 29.98 ± 3.31%; *p* = 0.658). Sex distribution did not differ significantly between groups (*p* = 0.268), and all enrolled calves received their first colostrum feeding within 30 min after birth.

**Table 1:** Baseline characteristics of control and hyoscine N-butylbromide-treated neonatal Jersey calves.

Characteristic	Control (*n* = 6)	Hyoscine N-butylbromide (*n* = 12)	*p*-value
Maternal colostrum Brix (%)	29.22 ± 3.58	29.98 ± 3.31	0.658
Sex, *n* (%)			0.268
Male	3 (50.0)	2 (16.7)	
Female	3 (50.0)	10 (83.3)	
First colostrum feeding within 30 min, *n* (%)	6 (100)	12 (100)	—

Maternal colostrum Brix values are presented as mean ± SD, and categorical variables as n (%). No statistical comparison was performed for the timing of first colostrum feeding because all calves received their first feeding within 30 min after birth. SD, standard deviation.

The effects of hyoscine N-butylbromide administration on passive transfer indicators and selected serum biochemical variables are presented in Table 2. The distribution of individual observations for serum Brix values, IgG, total protein, GGT, and glucose concentrations is shown in Figure 1.

**Table 2:** Serum Brix values and selected serum biochemical variables in control and hyoscine N-butylbromide-treated neonatal Jersey calves.

Variable	Control (*n* = 6)	Hyoscine N-butylbromide (*n* = 12)	*p*-value
48 h serum Brix (%)	9.53 ± 0.7	10.96 ± 0.81	**0.002**
48 h serum IgG (mg/dL)	2,175 ± 1,150	3,243 ± 794	**0.034**
48 h serum total protein (g/dL)	6.34 ± 1.23	7.06 ± 0.56	0.217
48 h serum GGT (U/L)	657 ± 421	882 ± 392	0.279
48 h serum glucose (mg/dL)	81.80 ± 19.6	84.10 ± 26.9	0.859

Values are presented as mean ± SD. IgG, immunoglobulin G; GGT, gamma-glutamyl transferase. *p*-values were obtained using independent-samples *t*-tests, except for serum total protein, which was analyzed using Welch’s independent-samples *t*-test. Statistical significance was declared at *p* < 0.05.

**Figure 1:**
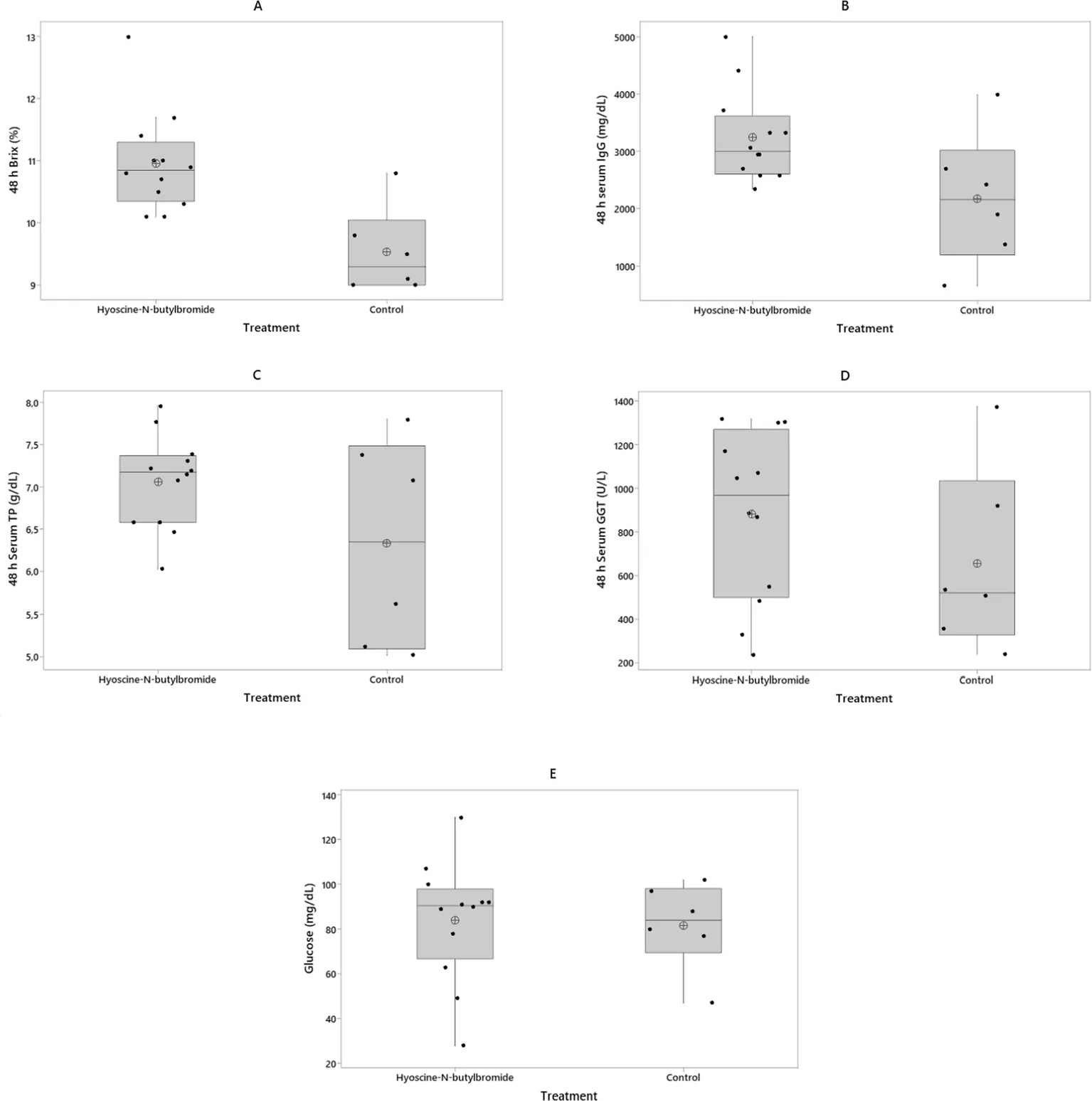
Distribution of serum Brix values and serum biochemical variables in control and hyoscine N-butylbromide-treated neonatal Jersey calves. Boxplots show the median and interquartile range, with whiskers indicating the data range. Individual observations are shown as points, and the mean is indicated by a separate symbol. **(A)** 48 h serum Brix (%); **(B)** 48 h serum IgG concentration (mg/dL); **(C)** 48 h serum total protein concentration (g/dL); **(D)** 48 h serum GGT activity (U/L); and **(E)** 48 h serum glucose concentration (mg/dL).

Serum Brix values were higher in hyoscine N-butylbromide-treated calves compared with the control group at 48 h (10.96 ± 0.81 vs. 9.53 ± 0.7%; *p* = 0.002).

Serum IgG concentrations at 48 h were also higher in hyoscine N-butylbromide-treated calves than in control calves (3,243 ± 794 vs. 2,175 ± 1,150 mg/dL; *p* = 0.034).

Serum total protein concentrations did not differ between groups (7.06 ± 0.56 vs. 6.34 ± 1.23 g/dL; *p* = 0.217). Similarly, serum GGT activity was not different between hyoscine N-butylbromide-treated and control calves (882 ± 392 vs. 657 ± 421 U/L; *p* = 0.279).

Serum glucose concentrations did not differ between groups (84.10 ± 26.9 vs. 81.80 ± 19.6 mg/dL; *p* = 0.859).

## 4 Discussion

In the present study, administration of hyoscine N-butylbromide prior to the first colostrum feeding was associated with higher serum IgG concentrations and serum Brix values in neonatal calves, suggesting improved passive transfer indicators. These findings are consistent with the hypothesis that modulation of gastrointestinal motility may influence the systemic availability of colostral immunoglobulins.

The observed increase in serum IgG concentrations in hyoscine N-butylbromide-treated calves suggests that gastrointestinal transit dynamics may contribute to macromolecule absorption. One possible explanation is that hyoscine N-butylbromide-induced alterations in gastrointestinal motility may modify luminal transit and the contact time between colostral IgG and the intestinal epithelium, thereby potentially influencing interaction with enterocytes and FcRn-associated transport mechanisms (11, 12, 24). A slower abomasal emptying rate could theoretically prolong the availability of colostral immunoglobulins for intestinal absorption (15). However, this mechanism remains speculative, as effective intestinal absorption also depends on coordinated peristaltic and segmental activity, and excessive gastrointestinal hypomotility could potentially impair luminal transit rather than enhance absorption. Because abomasal emptying and intestinal motility were not directly measured in the present study, the observed differences in passive transfer indicators cannot be attributed to a specific gastrointestinal mechanism. The variability in serum IgG concentrations observed in the present study is consistent with previous findings indicating that passive transfer efficiency may differ among calves despite standardized colostrum management (23).

The higher serum Brix values observed in treated calves at 48 h further support greater systemic availability of colostral proteins, consistent with the use of serum-based indicators for assessing passive transfer status in dairy calves (25). Given the strong correlation reported between serum Brix and IgG concentrations, these findings are consistent with previous reports validating Brix refractometry as a reliable indirect indicator of passive transfer status under field conditions (21, 26, 27). The parallel increase in serum Brix values and serum IgG concentrations supports the interpretation that hyoscine N-butylbromide administration was associated with enhanced passive transfer indicators, although the underlying mechanism cannot be confirmed without direct measurement of gastrointestinal motility.

In contrast, serum total protein concentrations did not differ between groups. This may be explained by the fact that total protein reflects a composite of multiple circulating protein fractions, including albumin and other non-immunoglobulin proteins, and is therefore less specific than serum IgG or serum Brix for detecting differences in passive transfer under standardized feeding conditions (21). Consequently, total protein may lack sensitivity to detect moderate differences in immunoglobulin absorption under controlled colostrum management conditions.

Similarly, serum GGT activity did not differ between groups despite differences in IgG concentrations. Although GGT is commonly used as an indicator of colostrum intake, its variability is influenced by factors such as colostral enzyme concentration and individual absorption kinetics (28). Therefore, the absence of a significant between-group difference in GGT activity is more appropriately interpreted as reflecting its role as a qualitative indicator of colostrum ingestion rather than as a quantitative surrogate for serum IgG concentration. The high GGT activities observed in both groups are consistent with successful colostrum intake, while biological variation in colostral GGT concentration among dams may also contribute to the observed variability. Previous studies have also emphasized that indirect indicators of passive transfer, including serum enzymes and metabolites, may be influenced by sampling time and physiological variation during the neonatal period (29).

Serum glucose concentrations were not affected by hyoscine N-butylbromide administration, indicating that treatment was not associated with detectable alterations in glucose homeostasis in neonatal calves. Colostrum is a critical source of energy required for thermoregulation, metabolic activation, and survival in newborn calves (30, 31). The maintenance of glucose concentrations within physiological ranges in both groups suggests that the intervention did not produce measurable disruption in early postnatal energy metabolism. This finding is important because the observed differences between groups were evident for passive transfer indicators, particularly serum IgG and serum Brix values, but not for the measured metabolic variables.

Recent studies have continued to explore nutritional and management-related factors affecting colostral immunoglobulin transfer, neonatal immune responses, gastrointestinal health, and calf welfare beyond conventional colostrum management practices (32–35). In this context, the present findings suggest that modulation of gastrointestinal motility may represent an adjunctive research avenue rather than a replacement for established colostrum management principles.

Nevertheless, several limitations should be considered when interpreting the findings of the present study. The relatively small sample size and unequal group distribution may limit the broader generalizability of the results. An *a priori* sample size calculation was not performed. Although the study population was deliberately restricted to a relatively homogeneous group of eligible calves to reduce biological variability, the modest sample size may have limited the precision of some estimates, particularly for outcomes in which no significant between-group differences were observed. Larger prospective studies are warranted to confirm these findings and provide more precise effect estimates. Because group allocation was not fully randomized or concealed, selection bias cannot be completely ruled out. In addition, as the final six eligible calves were enrolled later and assigned to the treatment group, potential temporal confounding should also be considered. However, the relatively narrow enrollment period and similar management and environmental conditions may have limited the potential effect. The study population was intentionally standardized by including only Jersey calves born from third-lactation cows within a narrow seasonal period in order to minimize biological variability associated with breed, parity, and environmental factors. Although this approach reduced the number of eligible animals, it increased experimental consistency and reduced potential confounding effects. In addition, the study was conducted on a single commercial dairy farm under controlled management conditions using high-quality colostrum, which may not fully reflect the variability encountered under different field conditions. Another limitation is that intestinal motility was not measured directly; therefore, the proposed mechanism underlying the observed differences in passive transfer remains indirect. In addition, gastrointestinal tolerance and clinical safety were not systematically evaluated using predefined clinical endpoints. Pharmacological reduction of gastrointestinal motility in neonatal calves may theoretically impair luminal clearance and favor the retention of bacteria and their products within the gastrointestinal tract. In the present study, no overt clinical complications, including delayed meconium passage or abdominal distension, were observed in the treated calves. However, these observations were not obtained using a predefined clinical safety protocol, and the study was not designed to establish the safety profile of hyoscine N-butylbromide in neonatal calves. Therefore, future studies should include systematic clinical monitoring of gastrointestinal tolerance alongside direct assessment of gastrointestinal motility. Accordingly, further studies involving larger populations, different management conditions, and direct measurements of gastrointestinal motility are warranted to confirm the broader applicability and physiological basis of these findings.

Hyoscine N-butylbromide administration before colostrum feeding was associated with higher serum IgG concentrations and serum Brix values in neonatal calves, without detectable differences in serum total protein, GGT, or glucose concentrations. Although these findings support a possible relationship between gastrointestinal transit dynamics and passive immunity acquisition, further studies involving larger populations, different management conditions, and direct assessment of intestinal motility are needed to confirm the broader applicability and physiological basis of this approach.

## Data Availability

The raw data supporting the conclusions of this article will be made available by the authors, without undue reservation.
